# Intraoperative wound irrigation to prevent surgical site infection after laparotomy (IOWISI): study protocol for a randomized controlled trial

**DOI:** 10.1186/s13063-017-2154-6

**Published:** 2017-09-04

**Authors:** Tara C. Mueller, Ulrich Nitsche, Victoria Kehl, Rebekka Schirren, Beate Schossow, Ruediger Goess, Helmut Friess, Daniel Reim, on behalf of the IOWISI Study Group

**Affiliations:** 1 Department of Surgery, Klinikum Rechts der Isar, Technische Universität München, Ismaninger Straße 22, 81675 Munich, Germany; 2 Institute for Medical Statistics and Epidemiology, Klinikum Rechts der Isar, Technische Universtität München, Ismaninger Straße 22, 81675 Munich, Germany; 3 Munich Centre for Clinical Trials (Münchner Studienzentrum), Technische Universtität München, Ismaninger Straße 22, 81675 Munich, Germany

**Keywords:** Surgical site infection, Abdominal surgery, Visceral surgery, Laparotomy, Intraoperative wound irrigation, Polyhexanide, Randomized controlled trial

## Abstract

**Background:**

Postoperative surgical site infection (SSI) is one of the most common hospital infections and contributes substantially to postoperative morbidity and mortality. In addition, SSIs dramatically increase the treatment cost and length of hospital stay. Following visceral surgery by laparotomy, SSI rates are especially high (14–25%). Therefore, measures to prevent SSI in this field are urgently needed. Prophylactic intraoperative wound irrigation (IOWI) of the subcutaneous soft tissue before skin closure hypothetically represents an easy and economical option to reduce SSI rates and is already frequently used in clinical practice. However, there are currently no definite recommendations on the use of IOWI since high-level evidence supporting its use is lacking. Consequently, clinical practice varies widely. Antiseptic polyhexanide (PHX)-based solutions are approved for soft-tissue wound irrigation in surgery but have not been specifically evaluated in randomized clinical trials for the prevention of SSI following laparotomy for visceral surgery.

**Methods/design:**

The IOWISI trial is a multicentre, randomized, observer- and patient-blinded clinical trial with three parallel treatment groups, comparing IOWI with a 0.04% PHX solution to no irrigation (test 1) or saline (test 2) before skin closure after laparotomy for visceral surgery (contamination level II–IV). The primary endpoint of the trial is the SSI rate within 30 days postoperatively. Statistical analysis of the primary endpoint measure will be based on the intention-to-treat population. The global level of significance is set at 2.5% for test 1 and 5% for test 2 and the sample size (*n* = 540) is determined to assure a power of 94% (test 1) and 85% (test 2).

**Discussion:**

The IOWISI trial will provide high-level evidence as a basis for clinical recommendations regarding the use of IOWI with PHX or saline and will potentially impact on future clinical guidelines and practice. The pragmatic trial design guarantees high external validity.

**Trial registration:**

Registered at the German Clinical Trials Register, DRKS00012251. Registered on 3 July 2017.

**Electronic supplementary material:**

The online version of this article (doi:10.1186/s13063-017-2154-6) contains supplementary material, which is available to authorized users.

## Background

### Rationale

Postoperative surgical site infection (SSI) is one of the most common hospital infections. SSI rates are especially high following visceral surgery by laparotomy. Depending on the level of intraoperative contamination, recent high-level randomized controlled trials (RCTs) with standardized SSI definitions report postoperative SSI rates between 14.5% and 25.0% [1–3]. Studies have shown an increase of 6–24 days in the mean length of hospital stay if SSI occurs [4]. In addition to the risk and discomfort for the patient, SSIs dramatically increase the cost of treatment. In Germany, postoperative SSIs account for approximately 1 million extra days of hospitalization and an additional cost of around € 3 billion per year [5]. Prophylactic intraoperative wound irrigation (IOWI) of the subcutaneous and deep soft tissue before skin closure with saline or antiseptic solutions hypothetically represents an easy and economical option to reduce SSI rates and is already frequently used in clinical practice [6, 7]. However, the latest official guidelines for the prevention of SSI by the Centres for Disease Control and Prevention (CDC) [8] and the World Health Organization (WHO) [9] conclude that IOWI with saline is not efficient and IOWI with diluted polyvinylpyrrolidone-iodine (PVP-I) solutions may have a potential benefit in preventing SSI. However, due to the low level of underlying evidence these recommendations are conditional [8, 9]. In contrast, the clinical guidelines of the British National Institute for Health and Clinical Excellence (NICE) from 2008 state that the efficacy of IOWI is unproven for any irrigation solution and that its use should be avoided to prevent potential tissue toxicity and systemic side effects of PVP-I and other antiseptics [10]. However, all of these recommendations are based on a small number of unstandardized and heterogeneous RCTs evaluating different types of surgery (abdominal, as well as orthopaedic and neurosurgical procedures) and different types and concentrations of irrigation solutions.

Antiseptic polyhexanide (PHX)-based solutions are approved for prophylactic intraoperative soft tissue wound irrigation and have been shown to be tissue tolerable without being resorbed [11, 12]. Therefore, side effects such as allergic reactions are extremely rare. PHX inhibits microbial attachment to wound surfaces through an interaction with the bacterial membrane. In vitro data show a superiority of PHX over PVP-I against a broad microbial spectrum, including multi-resistant strains [13]. However, there are no recommendations on IOWI with PHX in any of the current guidelines on SSI prevention [8–10].

### Previous trials

Even though the literature evaluating SSI prevention is substantial, high-level evidence to guide decisions on the use of IOWI with saline or antiseptics remains scarce. Our research group previously performed a large-scale meta-analysis of 41 RCTs on IOWI with saline, PVP-I, or antibiotic irrigation solutions, exclusively in abdominal surgery. The results of this analysis show a risk reduction of 46% in the treatment group (IOWI with any irrigation solution) [14]. The SSI incidence was 9% in the treatment group compared to 16% in the untreated group (*p* < 0.05). However, the majority of included trials were out of date (1970–1990) and most of them revealed a high risk of bias mainly because of insufficient data reporting or methodological flaws. In addition, interventions, follow-up times, and definitions of SSI varied widely between those trials. Furthermore, neither the WHO nor the NICE guidelines recommend the use of topical antibiotic solutions for this indication anymore [9, 10].

The only standardized RCT comparing IOWI with saline irrigation versus no irrigation after open appendectomies was published in 2000 and found a reduction of SSI from 25% to 8.7% in the saline irrigation group [15].

Recently, PHX-based antiseptic solutions have been successfully and widely used in orthopaedic and trauma surgery [11, 16]. In a longitudinal cohort study, irrigation of traumatic contaminated soft tissue wounds with PHX showed a reduction of the SSI rate of almost 75% compared to irrigation with Ringer’s solution [16, 17]. One pilot study investigated differences in SSI rates for “short” versus “long” IOWI with PHX 0.04% solution in abdominal surgery in 97 patients [18]. In the experimental group the subcutaneous tissue was irrigated through the aponeurosis prior to the initial incision and the wound margins were protected with PHX-soaked abdominal cloths during the whole procedure. After closing the abdominal fascia, wounds were again irrigated with PHX (“long”). In the retrospective control group, wounds were irrigated with PHX only before skin closure (“short”). No significant differences in SSI rates were observed between the two groups. The overall SSI rate in this single-centre study was comparable to observations of other recent trials (19.6%) [18]. This might be explained by the fact that wounds were subsequently rinsed with saline solution after PHX irrigation (see Discussion).

### Objective

The IOWISI trial aims to investigate if IOWI with PHX solution (0.04%) or saline (NaCl 0.9%) can reduce the rate of postoperative SSI within 30 days (according to the CDC definition [19]; Table 1) after laparotomy for visceral surgery. The results of the IOWISI trial will provide high-level evidence for future clinical recommendations regarding the use of IOWI in visceral surgery by laparotomy and provide the participating patients the opportunity of a potentially improved treatment.

**Table 1:** Definition and classification of surgical site infection (SSI) [19]

Superficial incisional SSI	Infection occurs within 30 days after the operation and infection involves only skin or subcutaneous tissue of the incision and at least *one* of the following: • Purulent drainage, with or without laboratory confirmation, from the superficial incision. • Organisms isolated from an aseptically obtained culture of fluid or tissue from the superficial incision. • At least one of the following signs or symptoms of infection: pain or tenderness, localized swelling, redness, or heat *and* superficial incision is deliberately opened by surgeon, *unless* incision is culture-negative. • Diagnosis of superficial incisional SSI by the surgeon or attending physician. Notes: Do *not* report the following conditions as SSI: • Stitch abscess (minimal inflammation and discharge confined to the points of suture penetration).
Deep incisional SSI	Infection occurs within 30 days after the operation and the infection appears to be related to the operation and infection involves deep soft tissues (e.g. fascial and muscle layers) of the incision and at least *one* of the following: • Purulent drainage from the deep incision but not from the organ/space component of the surgical site. • A deep incision spontaneously dehisces or is deliberately opened by a surgeon when the patient has at least one of the following signs or symptoms: fever (>38 °C), localized pain, or tenderness, unless site is culture-negative. • An abscess or other evidence of infection involving the deep incision is found on direct examination, during reoperation, or by histopathologic or radiologic examination. • Diagnosis of a deep incisional SSI by a surgeon or attending physician. Notes: • Report infection that involves both superficial and deep incision sites as deep incisional SSI. • Report an organ/space SSI that drains through the incision as a deep incisional SSI.
Organ/space SSI	Infection occurs within 30 days after the operation and the infection appears to be related to the operation and infection involves any part of the anatomy (e.g. organs or spaces), other than the incision, which was opened or manipulated during an operation and at least *one* of the following: • Purulent drainage from a drain that is placed through a stab wound into the organ/space. • Organisms isolated from an aseptically obtained culture of fluid or tissue in the organ/space. • An abscess or other evidence of infection involving the organ/space that is found on direct examination, during reoperation, or by histopathologic or radiologic examination. • Diagnosis of an organ/space SSI by a surgeon or attending physician.

## Methods/design

### Trial sites

The IOWISI trial will be conducted in at least 10 surgical departments (university and community hospitals), all of which are members of the German surgical trial network (CHIR-*Net*) and have previously participated in multicentre trials. All of the study personnel involved in the trial require training according to the International Conference on Harmonisation of Technical Requirements for Registration of Pharmaceuticals for Human Use – Good Clinical Practice (ICH-GCP) and will be instructed in all trial-specific procedures before initiation of the trial, conforming to German Drug Law (*Arzneimittelgesetz* (AMG) [20]) and GCP regulations [21]. After training by the principal investigator, the leading surgeon of the operating team will perform the intervention.

### Trial population and eligibility criteria

All adult patients (≥18 years) scheduled for visceral surgery by midline or transverse laparotomy (elective and emergency) will be eligible if they are able to understand the extent and nature of the IOWISI trial and provide written informed consent to participate. Exclusion criteria were defined as: a) pregnant or breast-feeding women; (b) known hypersensitivity/allergy to PHX; (c) American Society of Anesthesiologists (ASA) score > 3; (d) critical medical condition of emergency patients precluding informed consent or sufficient time to reflect on the decision to participate in the trial; (e) inability to give/understand informed consent or to attend follow-up visits; (f) clean/Class I procedures according to the CDC classification (Table 2) or surgery without opening of the abdominal cavity; (g) laparoscopic surgery; (h) previous abdominal surgery within the last 30 days or planned re-laparotomy within the next 30 days; (i) patients with severe immunosuppression; (j) presence of concurrent abdominal wall infections; (k) preoperative systemic antibiotic therapy within 5 days prior to surgery (except emergency antibiotic treatment due to septic peritonitis after admission to the hospital; routine intraoperative antibiotic prophylaxis is allowed); (l) participation in another clinical trial that interferes with the primary or secondary outcomes of this trial.

**Table 2:** Classification of wound contamination levels according to CDC [19]

Class I/clean	These are uninfected operative wounds in which no inflammation is encountered and the respiratory, alimentary, genital, or uninfected urinary tracts are not entered. In addition, clean wounds are primarily closed and, if necessary, drained with closed drainage. Operative incisional wounds that follow non-penetrating (blunt) trauma should be included in this category if they meet the criteria. Laparoscopic surgeries, surgeries involving the skin (such as biopsies), eye, or vascular surgeries are good examples.
Class II/clean-contaminated	An operative wound in which the respiratory, alimentary, genital, or urinary tracts are entered under controlled conditions and without unusual contamination. Specifically, operations involving the biliary tract, appendix, vagina, and oropharynx are included in this category, provided no evidence of infection or major break in technique is encountered.
Class III/contaminated	Open, fresh, accidental wounds. In addition, operations with major breaks in sterile technique (e.g. open cardiac massage) or gross spillage from the gastrointestinal tract, and incisions in which acute, non-purulent inflammation is encountered are included in this category. Contaminated wounds are also created when an outside object comes in contact with the wound (e.g. a bullet, knife blade, or other pointy object).
Class IV/dirty-infected	Old traumatic wounds with retained devitalized tissue and those that involve existing clinical infection or perforated viscera or a foreign object lodged in the wound or any wound that has been exposed to pus or faecal matter. This definition suggests that the organisms causing postoperative infection were present in the operative field before the operation.

*CDC* Centres for Disease Control and Prevention

### Sample size

For statistical significance 540 patients have to be recruited, 230 patients in the PHX and the saline group, respectively, and 80 patients in the control group (see Statistical methods).

### Type of trial

This is a randomized, controlled, observer- and patient-blinded, multicentre, surgical trial with three parallel study groups, phase IIIb according to German Drug Law (*AMG*).

### Recruitment and trial timeline

**Fig. 1:**
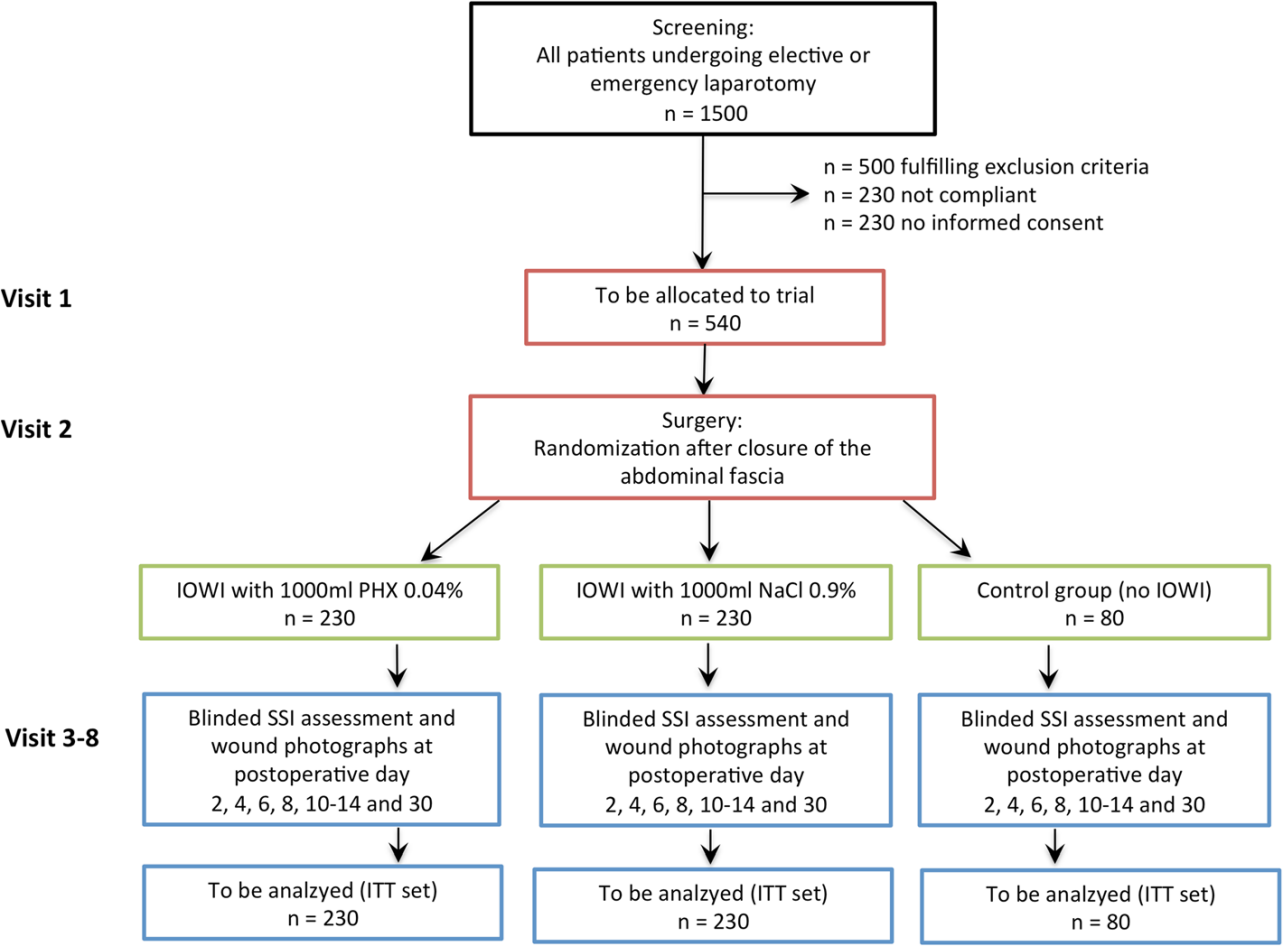
Flow chart of the IOWISI trial. *IOWI* intraoperative wound irrigation, *PHX* polyhexanide, *SSI* surgical site infection, *ITT* intention-to-treat

**Fig. 2:**
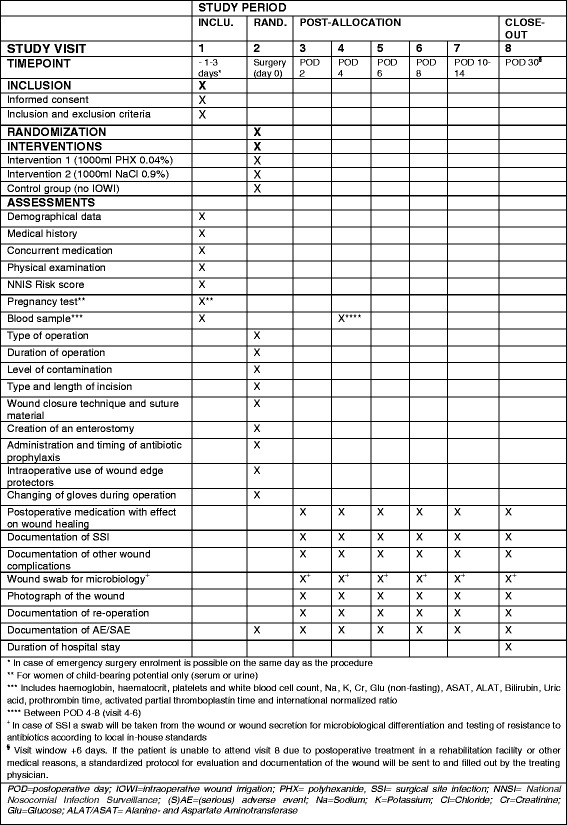
SPIRIT figure for the IOWISI trial (according to SPIRIT 2013 [22]). *AE* adverse event, *ALAT* alanine aminotransferase, *ASAT* aspartate aminotransferase, *Cr* creatinine, *Glu* glucose, *IOWI* intraoperative wound irrigation, NNIS National Nosocomial Infection Surveillance, *PHX* polyhexanide, *POD* postoperative day, *SAE* serious adverse event, *SSI* surgical site infection, *ITT* intention-to-treat

Only surgical departments with adequate patient numbers will be included in the trial to assure the target sample size. The recruitment period is set at 27 months (first patient in to last patient out, 28 months). Figure 1 shows the trial flow scheme and Fig. 2 (SPIRIT figure) shows the schedule of enrolment, interventions, and assessments according to the diagram of the SPIRIT 2013 statement [22]. The SPIRIT checklist for this study protocol can be found in Additional file 1.

### Randomization and blinding

Prior to inclusion in the trial, a GCP-certified investigator will perform the screening and collect detailed information from the patients and will obtain their written informed consent. An online tool of the Munich Centre of Clinical Trials (*Münchner Studien Zentrum* (MSZ)) will be used to generate the allocation sequence. This online tool uses pre-defined randomization lists, which will be created block-wise at the Institute of Medical Statistics and Epidemiology (*Technische Universität München* (TUM)) and will be stratified by study centre and level of contamination of the surgical procedure (class II–IV; Table 2).

A member of the study team will perform randomization during surgery after closure of the abdominal fascia is completed and will inform the surgical team of the assigned treatment. To assure balanced group sizes in the course of the accrual, a block-wise randomization is applied. Baseline characteristics of the patient and day of randomization must be documented on the printed randomization sheets. Subsequently, randomization sheets must be stored away from the patient records and trial documents to ensure blinding. The surgical team cannot be blinded, as no irrigation is used in the control group. However, patients, outcome assessors, and the trial statistician will be blinded for the trial intervention. The primary endpoint (SSI up to postoperative day 30) will be assessed clinically by a member of the local study team who will neither be part of the surgical team that performs the intervention nor have access to the randomization results in order to comply with observer-blinding. In addition, wound photographs will be taken at each study visit and evaluated via a central database by independent outcome assessors at spatially separated participating study sites. Unblinding of the local principal investigator is permissible if significant hazards for subjects’ safety or welfare occur.

### Interventions

#### Experimental interventions

Preoperative skin preparation, application of antibiotic prophylaxis, and the surgical procedure will be performed according to local standard procedures. After closure of the abdominal fascia, patients will be randomized to one of the following groups.

In the experimental group 1, the subcutaneous soft tissue will be irrigated with 1000 ml of a 0.04% PHX solution, which is the recommended concentration for surgical wound irrigation according to the medicinal product's professional information. The whole surface of the wound will be carefully rinsed with the irrigation solution and the excess fluid will be removed by suction. Debris and blood clots should be removed from the wound using irrigation/suction. The wound is not to be rubbed dry with abdominal cloths, but left moistened with the irrigation solution to ensure sufficient contact time for PHX to assure the desired antiseptic effect. After irrigation with PHX the wound will not be irrigated with saline again. Since PHX is a cation-active substance, it is not compatible with anionic organic substances (e.g. lactate). Furthermore, the combination of PHX with PVP-I products has to be avoided.

In the experimental group 2, the same intervention will be performed using 1000 ml of saline solution (NaCl 0.9%).

After irrigation of the wound, the skin closure and sterile dressing will be performed according to local standards, without any further wound-related procedure. PHX and saline solutions are to be purchased, stored, and distributed according to the respective standard operating procedures of the trial centres. If the operating surgeon decides that incomplete closure of the wound and/or any other wound-related procedure after the study intervention (e.g. negative pressure treatment) is necessary for the benefit of the patient, the patient will have to be excluded from the trial.

#### Control intervention

In the control group, wounds will not be surgically irrigated according to the NICE guideline recommendations [10].

### Permitted and not permitted medication(s)/treatment(s)

No additional study-specific treatments will be performed within the trial. Antibiotic treatment 5 days prior to surgery is an exclusion criterion. Preoperative antibiotic treatment due to septic peritonitis is allowed, but has to be recorded in the electronic case report form (eCRF). Application and timing of routine intraoperative antibiotic prophylaxis will be recorded in the eCRF. If indicated for medical reasons, all kinds of medication are permitted during the trial. Postoperative administration of antibiotics or medication with adverse effects on wound healing (e.g. immunosuppressive agents) will be recorded in the eCRF.

### Risks

No additional risks for study patients are anticipated, since IOWI with PHX or saline, as well as the control intervention, represent clinically established standard methods. PHX 0.04% irrigation solution is approved for surgical wound irrigation of soft tissue wounds in Germany. The study will be planned, conducted, and analysed according to all relevant national and international rules and regulations (AMG [20], ICH-GCP [21], Declaration of Helsinki [23]). The safety of PHX solutions has been demonstrated in marketing studies. However, adverse effects may only be expected in the improbable event of accidental contamination of the respective irrigation solutions or in case of unknown hypersensitivity to PHX. The potential benefits of a reduced risk of SSI outweigh the mentioned negligible potential adverse effects of PHX. The safety of the subjects is ensured by regular study visits, enforcing GCP guidelines. Furthermore, each patient will be provided with insurance coverage for any potential harm from trial participation.

### Outcome measures

The primary efficacy endpoint measure of the trial is the incidence of SSI according to the CDC definition and classification within 30 days after surgery (Table 1). If the patient’s wound cannot be evaluated exactly on postoperative day 30, a clinical evaluation up to postoperative day 36 will be allowed. However, only SSIs that occurred up to day 30 will be counted as postoperative SSI, conforming to the CDC definition. In addition, the following outcome measures have been defined as secondary endpoint measures and will be determined by the unit given in parentheses: a) duration of hospital stay (in days); b) 30-day rate of reoperation in all groups (%); c) 30-day rate of wound complications other than SSI in all groups (%); d) 30-day mortality in all groups (%); and e) 30-day rate of postoperative adverse events/serious adverse events (AE/SAE) in all groups (%). All AE/SAEs that are surgical complications will be additionally classified according to the Clavien Dindo classification of surgical complications (Table 3) [24].

**Table 3:** Clavien Dindo classification of surgical complications [24]

Grade	Definition
Grade I	Any deviation from the normal postoperative course without the need for pharmacological treatment or surgical, endoscopic, and radiological interventions
Grade II	Allowed therapeutic regimens are: drugs as antiemetics, antipyretics, analgetics, diuretics, electrolytes, and physiotherapy. This grade also includes wound infections opened at the bedside
Grade III	Requiring pharmacological treatment with drugs other than such allowed for grade I complications. Blood transfusions and total parenteral nutrition are also included. Requiring surgical, endoscopic, or radiological intervention
Grade IIIa	Intervention not under general anaesthesia
Grade IIIb	Intervention under general anaesthesia
Grade IV	Life-threatening complication (including CNS complications) requiring IC/ICU management
Grade IVa	Single organ dysfunction (including dialysis)
Grade IVb	Multiorgan dysfunction
Grade V	Death of a patient
Suffix “d”	If the patient suffers from a complication at the time of discharge, the suffix “d” (for “disability”) is added to the respective grade of complication. This label indicates the need for a follow-up to fully evaluate the complication.

*CNS* central nervous system, *IC* intermediate care, *ICU* intensive care unit

### Data collection and management

The applicable local regulations of data privacy protection will be followed. Prior to inclusion, patients will be informed that any patient-related data and materials will be appropriately pseudonymized and that these data may be used for analysis and publication purposes, pursuant to GCP regulations. All trial data will be recorded in eCRFs in a central database of the MSZ (MACRO™ version 3, Microsoft SQL Database, using Microsoft Internet Explorer version 6 or higher). After inclusion, baseline data (demographical data, medical history including history of SSI and history of radio/chemotherapy, concurrent medication, body mass index (BMI), ASA score, smoking/alcohol use, length of preoperative hospital stay, National Nosocomial Infection Surveillance (NNIS) risk score) will be documented accordingly. A physical examination will be performed and a blood sample will be taken (see Fig. 2 for included parameters). In the case of women of child-bearing potential, a pregnancy test will be additionally performed. Randomization will take place at the end of surgery after closure of the abdominal fascia when the level of contamination is definitely determined by the surgeon. Documented parameters of the surgical procedure include the administration and timing of antibiotic prophylaxis, the type and duration of the respective surgical procedure, the level of contamination according to CDC classification after closure of the abdominal fascia, the creation of an enterostomy, the intraoperative use of wound edge protectors and prophylactic changing of gloves during the operation, the wound closure technique, and the used suture material. Postoperatively, the local study personnel will perform six study visits. Wounds will be examined according to the CDC criteria for SSI, photographs will be taken at each visit, and between visits 4 and 6 one more routinely obtained blood sample will be analysed. In case of SSI, an additional microbiological swab will be taken from the wound. Postoperative medication with adverse effects on wound healing or antibiotics will be recorded in the eCRF.

To promote complete follow-up, patients that have already been discharged from the hospital by the time of the study visits will be supported in arranging transport and expenses necessary to ensure participant retention. If, however, the patient is unable to attend visit 8 due to postoperative treatment in a rehabilitation facility or other medical reasons, a standardized protocol for evaluation and documentation of the wound (including wound photograph) will have to be filled out by the treating physician and sent to the coordinating study site for independent evaluation.

At the end of the clinical study all study-relevant data must be archived for at least 10 years. Patient identification lists and patient files are retained in the respective study sites separately. The infrastructure and the personnel for the data management will be provided by the Data Management Centre at the MSZ, a member of the German Network of Coordinating Centres for Clinical Trials (*Koordinierungszentren für Klinische Studien* (KKS)). The eCRFs are checked for completeness, plausibility, and correctness at entry. The investigator is responsible for the accuracy and verifiability of each entry. Validating programmes as well as individual inspection of data through the MSZ will ensure completeness, validity, and plausibility. Each remaining question or missing response is documented through data clarification requests, to which the local investigator is obliged to respond as quickly as possible.

### Access to data and dissemination of results

Only the sponsor, personnel authorized by the sponsor, and the trial statistician will have access to the final dataset. After completion of the clinical study, the sponsor-delegated coordinating investigator will prepare a multicentre manuscript of the study results for publication in a reputable scientific journal. Authorship eligibility will be regulated in the Clinical Trial Agreement for all study sites. The publication of the principal results from any single-centre experience within the trial is not allowed until the preparation and publication of the multicentre results.

### Monitoring

Monitoring will be performed by the MSZ, an institution experienced in the monitoring of multicentre, surgical RCTs, in order to guarantee high quality of the study conduct and data retrieval. Monitoring will be carried out in accordance with ICH-GCP guidelines [21]. Monitors will visit all participating centres on a regular base, starting with an initiation visit at each site. Furthermore, close-out visits are planned for each centre. A monitoring manual describing the scope of the monitoring activity in detail will be provided. The monitor is authorized to compare trial documents and original data in adherence to data protection rights. The investigator provides direct access to patient’s documents/original source data to the monitor at any time. The monitor will also contact all participating centres and the sponsor on a regular basis. Furthermore, the sponsor and the competent health authorities have the right to audit/inspect the trial sites involved in the trial as part of quality assurance according to GCP regulations.

### Safety evaluation and reporting of adverse events

All AE/SAEs from the moment of randomization until the last study visit have to be assessed, documented, and reported by the local principal investigator or designated sub-investigator. SSIs and all other local wound complications and surgical complications will be documented as AE/SAE, as well as their severity and the consequent treatment according to the Clavien Dindo Classification (Table 3). SAEs have to be reported within 24 h and will be classified by intensity, outcome, and causality. All SAEs will be subject to a second assessment by a designated person who will be independent from the reporting investigator and the trial sponsor. Pursuant to AMG and GCP regulations, the ethics committee and the competent federal authority will be informed of all suspected unexpected serious adverse reactions (SUSARs) and all SAEs resulting in death or that are life threatening during the trial. In addition, an independent Data and Safety Monitoring Board will address patient safety and assess regular safety reports for any termination criteria.

### Statistical methods

The statistical analysis will be conducted by a group allocation-blinded statistician from the Institute for Medical Statistics and Epidemiology of the TUM, in line with the ICH-GCP guidelines [21]. For the statistical analysis, SAS software version 9.2 or higher will be used (SAS Institute Inc., Cary, NC, USA).

#### Sample size calculation

The sample size was calculated (nQuery Advisor software version 7.0, Statistical Solutions Ltd, Cork, Ireland) based on the primary endpoints of the study, assuming SSI rates of 2.2% in the PHX group (according to the trial by Roth et al. [17]), 8.7% in the saline group (according to the results of the trial by Cervantes-Sanchez et al. [15]) and 16.2% in the control group (according to results of the meta-analysis by Mueller et al. [14]). The global significance level was set to 5%. Since the PHX arm will be used twice for a comparison, the Bonferroni-Holm procedure was used to set the local alpha level to 2.5% for test 1 (PHX versus no irrigation) and to 5% for test 2 (PHX versus saline). If 230 patients are recruited in the PHX arm, 230 patients in the saline arm, and 80 patients in the control arm (a total of 540 patients), the two-sided Fisher exact test will have a power of 94% for test 1 and 85% for test 2 to detect differences between the treatment groups. The comparison of saline irrigation versus control is not included in the sample size calculation as it will not be analysed in a confirmatory manner; the low medical interest cannot justify the large increase in patient numbers.

#### Analysis population

The primary and secondary endpoints will be analysed on the intention-to-treat (ITT) set consisting of all patients randomized in the study independent of the intervention they receive (analysis ‘as randomized’). The safety analysis will be performed on the safety set consisting of all patients randomized into the study and assigned to the treatment group of their actual treatment.

#### Analysis of the primary endpoint measure

Wound irrigation with PHX solution will be tested for superiority over no irrigation (test 1) and irrigation with saline (test 2) with respect to the incidence of SSI within 30 days postoperatively using two Fisher exact tests with the following hypotheses:

Test 1: *H*_1_0_ : *π*_*P*_ = *π*_*N*_ versus *H*_1_*A*_ : *π*_*P*_ ≠ *π*_*N*_

Test 2: *H*_2_0_ : *π*_*P*_ = *π*_*S*_ versus *H*_2_*A*_ : *π*_*P*_ ≠ *π*_*S*_

where *π*_*P*_, *π*_*N*_, and *π*_*S*_ denote the incidence of SSI in the PHX, no irrigation, and saline groups, respectively. The tests will be performed as two-sided and with a global significance level of 5%. Using the Bonferroni-Holm adjustment, the local significance level will be 2.5% for test 1 and 5% for test 2.

#### Sensitivity of the primary endpoint measure

A dropout rate of 8–10% is expected in this study based on experience from similar trials previously conducted in the CHIR-*Net* [1]. The primary endpoint analysis will be based on all patients with complete SSI follow-up. In order to examine the sensitivity of the results, multiple imputations will additionally be used to estimate missing primary endpoint data.

#### Supportive analysis of the primary endpoint

Randomization will be stratified by study centre and level of contamination. Supportive analysis of the primary endpoint will be performed using a binary logistic regression model with dependent variable SSI and the covariates treatment group, study centre, and level of contamination. In addition, the following parameters might influence the outcome which is why they will be included as model covariates:

- Operation-related risk factors: (a) type of surgery; (b) duration of surgery; (c) use of wound-edge protectors; (d) intraoperative changing of gloves before skin closure; (e) presence of an enterostomy; (f) administration and timing of antibiotic prophylaxis; (g) NNIS risk score;
- Patient-related risk factors: (h) BMI; (i) ASA score; (j) age; (k) diabetes; (l) duration of preoperative hospital stay; (m) smoking; (n) alcohol abuse; (o) history of SSI; (p) history of radio- and/or chemotherapy.

Furthermore, surgical and patient characteristics will be monitored to identify other potential confounders and to allow adjustment for these variables.

#### Analysis of secondary endpoint measures

Secondary endpoints will be analysed by treatment arm on the ITT set using appropriate descriptive statistics. Any explorative statistical testing will be performed using a significance level of 5%.

#### Safety analysis

All AE/SAEs will be analysed with incidence rates by treatment group and according to severity. All AE/SAEs rated as related to the study treatment will be listed separately. For the comparisons between groups, the Chi-square test and the Fisher exact test will be used if appropriate.

### Withdrawals

Patients are free to withdraw from trial participation at their own request at any time and without giving reasons for their decisions. Withdrawals will be documented in the eCRF and in the patient’s medical record. However, all on-going SAEs must be followed up and documented until their final outcome can be determined. In addition, the investigator has the right to withdraw a patient from the study at any time. Reasons for withdrawal from the study may include but are not limited to the following: (a) any medical condition that the investigator or sponsor determines may jeopardize the patient’s safety if he or she continues in the study; (b) if it is discovered that a study subject is pregnant or may have been pregnant at the time of intervention. Patients who withdraw from the study will not be replaced.

### Stopping guidelines

The trial can be prematurely closed by the sponsor in consultation with the responsible biostatistician for the following reasons: (a) it appears that patient enrolment is unsatisfactory with respect to quality or quantity, or data recording is severely inaccurate or incomplete; (b) there is external evidence demanding a termination of the trial, e.g. indicating that the rate or severity of SAEs or morbidity in this trial poses a potential health hazard caused by the intervention in one of the trial groups. In case of premature closure, the ethics committee and federal authority must be informed.

### Registration

The trial protocol was registered at the German Clinical Trials Register (part of the WHO International Clinical Trials Registry Platform) under the trial number DRKS00012251 on 3 July 2017.

### Good clinical practice

The procedures set out in this trial protocol, pertaining to the conduct, evaluation, and documentation of this trial, are designed to ensure that all persons involved in the trial abide by GCP [21] and the ethical principles described in the current version of the Declaration of Helsinki [23]. The trial will be carried out in keeping with all federal and local legal and regulatory requirements.

## Discussion

Currently, the official recommendations on the prophylactic use of IOWI and clinical practice vary largely. The WHO Global Guidelines for the prevention of SSI (2016) as well as the updated CDC guidelines (2017) conclude that there is insufficient evidence to recommend IOWI with saline and that IOWI with diluted PVP-I could be considered but also not routinely recommended [9]. However, the level of underlying evidence is low and trials analysed for these guidelines do not solely focus on visceral surgery, but include all types of surgery (e.g. orthopaedic or neurosurgery) which differ substantially in SSI rates and causative microorganisms. Furthermore, PVP-I solutions are seen as controversial for this prophylactic indication due to possible systemic adverse effects and tissue toxicity. However, despite its unproven efficacy, most general and visceral surgeons currently use IOWI to prevent SSI [25].

Solutions containing the antiseptic agent PHX are approved for IOWI, and were shown to be tissue tolerable and effective in vitro and in vivo [8, 9]. However, PHX solutions are not mentioned in the WHO or other recent guidelines. Ultimately, data from high-quality RCTs are needed to resolve the scientific equipoise regarding application of IOWI with saline or antiseptics.

The IOWISI trial has a pragmatic, three-armed study design (PHX *versus* saline irrigation *versus* no irrigation) and shall be conducted in at least 10 centres within the German Surgical Trial Network (CHIR-*Net*). A multicentre approach, including hospitals of different care levels, was chosen to increase external validity. Internal validity and data quality assurance are established by adherence to the SPIRIT statement and GCP regulations regarding recruitment, training of study personnel, methods against bias, outcome reporting, and documentation.

All patients undergoing visceral surgery by laparotomy within 27 months will be screened for this trial. Broad inclusion criteria were applied to ensure rapid and sufficient recruitment of the target sample size.

After closure of the abdominal fascia according to local standards, laparotomy wounds will either be irrigated with PHX or saline. The volume of 1000 ml was chosen to be sure that large laparotomy wounds would be sufficiently irrigated. This was determined by the clinical experience of senior surgeons at our institution since, so far, no recommendations for the optimal volume of surgical wound irrigation exist. The same applies to the duration and technique of irrigation. However, the POLIS pilot trial compared “short” versus “long” irrigation with PHX and no differences in SSI rates were observed [18]. In vitro experiments show that a contact time of 10–15 min is required for PHX to act fully [12]. To ensure this, the irrigation technique will be standardized in the IOWISI trial. In addition, wounds will be left moistened with the PHX solution at the end of the irrigation and shall not be rubbed dry with abdominal cloths or rinsed with saline again.

The primary endpoint is the incidence of SSI 30 days postoperatively, according to the widely accepted CDC definition and classification (Table 1) [19]. Since many different SSI definitions have been proposed over the past decades, standardized reporting is crucial for the comparability of trials regarding SSI prevention.

The results of this pragmatic trial will provide high-level evidence for clinical recommendations regarding the use of IOWI with PHX or saline to prevent SSI after laparotomy, potentially impact future clinical guidelines and provide participating patients the opportunity of an improved treatment.

### Trial status

Recruitment is planned to start from 1 September 2017.

## Additional files

Additional file 1: SPIRIT 2013 checklist: recommended items to address in a clinical trial protocol and related documents. (PDF 105 kb)

Additional file 2: List of local ethical committees that approved the IOWISI study protocol. (DOCX 60 kb)

## Abbreviations

AE: Adverse event
AMG: *Arzneimittelgesetz*/German Drug Law
ASA: American Society of Anaesthesiologists
CDC: Centres for Disease Control and Prevention
eCRF: Electronic case report form
GCP: Good Clinical Practice
ICH: International Conference on Harmonisation of Technical Requirements for Registration of Pharmaceuticals for Human Use
IOWI: Intraoperative wound irrigation
ITT: Intention-to-treat
KKS: *Koordinierungszentren für Klinische Studien*/German Network of Coordinating Centres for Clinical Trials
MSZ: *Münchner Studienzentrum*/Munich Centre of Clinical Trials
NNIS: National Nosocomial Infection Surveillance
PHX: Polyhexanide
RCT: Randomized controlled trial
SAE: Serious adverse event
SSI: Surgical site infection
SUSAR: Suspected unexpected serious adverse reaction
TUM: *Technische Universität München* (Technical University of Munich)
WHO: World Health Organization
