# Supplementary material for: Intraoperative wound irrigation to prevent surgical site infection after laparotomy (IOWISI): study protocol for a randomized controlled trial
Source: Trials. 2017 Sep 4;18:410. doi: 10.1186/s13063-017-2154-6 (PMC5584516; doi:10.1186/s13063-017-2154-6)
Supplement: Supplementary file 2 — List of local ethical committees that approved the IOWISI study protocol. (DOCX 60 kb) [file 13063_2017_2154_MOESM2_ESM.docx]

| **Study centre** | **Ethics Committee** | **Reference number** |
| --- | --- | --- |
| Asklepios Clinic Langen | Ethik Kommission der Landesärztekammer Hessen | MC 75/2017 |
| University Hospital Mainz | Ethik Kommission der Landesärztekammer Reinlandpfalz | 837.185.17 |
| University Hospital Dresden | Ethik Kommission der medizinischen Fakultät “Carl Gustav Carus” der Technischen Universität Dresden | EK215062017 |
| University Hospital Düsseldorf | Ethik Kommission der medizinischen Fakultät der Heinrich-Heine-Universität | MC-942 |
| University Hospital Heidelberg | Ethik Kommission I der medizinischen Fakultät Heidelberg | ABmu-229/2017 |
| University Hospital Lübeck | Ethik Kommission Universität zu Lübeck | 17-149 |
| University Hospital Hamburg | Ethik Kommission der Ärztekammer Hamburg | MC-138/17 |
